# Opportunities for Improved Serodiagnosis of Human Tuberculosis, Bovine Tuberculosis, and Paratuberculosis

**DOI:** 10.1155/2012/674238

**Published:** 2012-06-06

**Authors:** Ashutosh Wadhwa, Graham J. Hickling, Shigetoshi Eda

**Affiliations:** Department of Forestry, Center for Wildlife Health, Wildlife and Fisheries, University of Tennessee Institute of Agriculture, Knoxville, TN 37996, USA

## Abstract

Mycobacterial infections—tuberculosis (TB), bovine tuberculosis (bTB), and Johne's disease (JD)—are major infectious diseases of both human and animals. Methods presently in use for diagnosis of mycobacterial infections include bacterial culture, nucleic acid amplification, tuberculin skin test, interferon-**γ** assay, and serology. Serological tests have several advantages over other methods, including short turn-around time, relatively simple procedures, and low cost. However, current serodiagnostic methods for TB, bTB and JD exhibit low sensitivity and/or specificity. Recent studies that have aimed to develop improved serodiagnostic tests have mostly focused on identifying useful species-specific protein antigens. A review of recent attempts to improve diagnostic test performance indicates that the use of multiple antigens can improve the accuracy of serodiagnosis of these mycobacterial diseases. Mycobacteria also produce a variety of species-specific nonprotein molecules; however, only a few such molecules (e.g., cord factor and lipoarabinomannan) have so far been evaluated for their effectiveness as diagnostic antigens. For TB and bTB, there has been recent progress in developing laboratory-free diagnostic methods. New technologies such as microfluidics and “Lab-on-Chip” are examples of promising new technologies that can underpin development of laboratory-free diagnostic devices for these mycobacterial infections.

## 1. Introduction

Mycobacterial infections are a leading cause of health concerns in humans and animals worldwide. *Mycobacterium tuberculosis* (MTB), *Mycobacterium bovis* (MB), and *Mycobacterium avium* subspecies *paratuberculosis* (MAP) are the causative agents of human tuberculosis (TB), bovine tuberculosis (bTB), and Johne's disease (JD), respectively. In 2009, more than 9 million cases of TB were reported, causing 1.8 million deaths [1]. Multidrug-resistant TB strains and coinfections of TB and HIV are emerging problems globally [2–4]. Despite much progress in eradicating bTB in developed countries, this disease is responsible for US$ 3 billion economic losses globally [5] and remains prevalent in some wild species [6, 7]. MAP is present in 68% of US dairy herds [8], with JD responsible for an annual $220 million economic loss to the US dairy industry [9].

 Control measures for these mycobacterial diseases revolve around understanding their epidemiology and improving treatment/vaccination protocols; however, a major bottleneck has been the lack of efficient diagnostic methods [2, 10–12]. Consequently, there would be much benefit to the development of rapid and accurate diagnosis of TB at point-of-care [3] (In this paper, point-of-care diagnosis is defined as diagnostic methods that can be conducted on-site (e.g. field, bed-side), with or without a requirement for laboratory facilities. Laboratory-free (lab-free) diagnosis is defined as point-of-care diagnostic methods that does not require any laboratory facility). Similarly, the most common current diagnostic test for bTB, the tuberculin skin test (TST), is not practical for controlling bTB in wild animals, so a lab-free diagnostic device would also be helpful in this context. Diagnosis of JD is currently conducted annually or biannually in diagnostic laboratories. If a lab-free diagnostic device became available, it would reduce the long time interval and cost of diagnosis. Thus, there would be a great value in lab-free diagnostic technologies for TB, bTB, and JD [13, 14].

Unfortunately, efficient lab-free diagnostic devices for these diseases are not yet available [14, 15]. Here, therefore, we briefly review currently available and recently developed diagnostic methods for these three mycobacterial diseases and highlight the potential benefits of lab-free diagnosis. Since serodiagnosis has been the most favored format for development of lab-free diagnostic method, we focus in this paper on methods of serodiagnosis over other diagnostic methods such as bacterial culture and nucleic acid amplification that are necessarily laboratory based.

## 2. Human Tuberculosis

### 2.1. Background

Human tuberculosis (TB) is caused primarily by MTB and occasionally by MB and *M. africanum *(in this paper we focus on MTB). TB is a leading cause of human morbidity and mortality throughout the world [16]. One-third of the world's population is infected by MTB [1], although only 5–10% of infected individuals develop an active, life-threatening form of the disease. In 2009, 9.4 million cases of TB were reported with 1.8 million deaths worldwide [1, 2, 17].

 Depending on the pathogenesis, infectivity, immune response, and effectiveness of treatment, TB can be divided into 3 major forms. The first is the active form of TB (TBA), which results in a rapid development of clinical signs in patients following contact with MTB. TBA develops in only 5% of individuals infected with MTB; the remainder develops a strong acquired immune response showing no clinical signs, termed latent TB (TBL) [18]. The third form is multidrug-resistant TB (MDRTB), which constitutes approximately 5% of TBAs [19]. MDRTB is caused by organisms resistant to, at least, isoniazid and rifampin [20]. The overall prevalence of MDRTB in developed nations is much lower than that in developing nations, but can be high in immigrant populations and among prisoners and immunocompromised individuals [21, 22]. During the past two decades, the emergence of HIV infection has led to the recognition that TB/HIV coinfection promotes both the reactivation of TBA from TBL and also the rapid progression of primary TB following recent exposure to MTB [23].

 Controlling TB depends on the following factors: case detection, treatment of individuals with TBA, improving anti-TB therapy to prevent resistance, identification of TBL, and better vaccination strategies for susceptible individuals [16]. All these factors would benefit from a better understanding of the epidemiology of the TB infection [21] and the development of more cost-effective, evidence-based approaches for its diagnosis [22]. Efficient diagnosis of TB is particularly important in third world nations that presently lack adequate diagnostic resources at primary health care centers. In these nations, TBL and MDRTB often remain undiagnosed, which facilitates further transmission.

 Presently, there are a number of alternative diagnostic approaches towards diagnosis of TB and of TB coinfection with other emerging infectious diseases; these are reviewed briefly here.

### 2.2. Imaging and Microscopic Techniques

Radiographic imaging is still widely used to diagnose TB; however, there are no definitive diagnostic patterns, so that the method can be used only for screening of TB cases. Further bacteriological examinations are required for confirmation [64, 65]. Smear microscopy of stained sputum or other clinical material is the most common test for TBA. This relatively inexpensive method can be carried out rapidly in low-resource settings; however, it lacks sensitivity and requires a large number of bacilli (5,000–10,000 organisms/sample) [64, 66] in the clinical specimen, which is often not the case in children, advanced-stage TBA patients, and individuals coinfected with HIV. Fluorescent microcopy is more sensitive, but its application is limited by high cost and by issues relating to the use of mercury vapor lamps in conventional fluorescent microscopes [67]. Nucleic acid amplification (NAA) assays have been found useful for diagnosis of TBA and MDRTB infections, as they have high specificity and sensitivity and can provide results within a few hours. Unfortunately, these assays are costly, require a laboratory with trained staff, and suffer from poor specificity under field conditions [64, 68, 69].

### 2.3. Bacterial Culture and Cell-Mediated Immune-Response-Based Testing

 Bacterial culture is considered the gold standard for TBA diagnosis, having close to 98% specificity, and is also useful in diagnosis of MDRTB. However, the bacterial culture method suffers from low sensitivity (26–42%), delayed results (6–8 weeks are required for culture growth), a need for trained personnel and culture facilities, and the high cost of the culture examination. The need for technical expertise can be particularly problematic in developing nations. Parsons et al. have recommended new technologies including urine antigen detection, assays based on volatile markers, bead-based, and flow-cytometric-based assays [3]—to help address these problems, but these assays await optimization and establishment of clinical utility.

The tuberculin skin test (TST)—based on detection of delayed-type hypersensitivity after an intradermal injection of purified protein derivative (PPD) extracted from heat killed MTB—has been in use for almost a century. The primary roles of TST are to identify TBL individuals and to monitor recent infection in high-risk groups. Some limitations of TST include a high frequency of false reactions, the need for a follow-up visit after 2-3 days of PPD inoculation, misleading results due to confounding factors (e.g., age, HIV infection, and infection with other mycobacterial species or cancer), and positive reactions in TBA patients [64, 67, 70]. Based on the identification of MTB-specific antigens using molecular techniques, detection of cell-mediated immune (CMI) response against MTB infection has improved the diagnosis of TB. These assays measure the production of cytokines (mainly interferon-gamma [IFN-*γ*]) produced by T cells of MTB-infected individuals. Initial IFN-*γ* assays were based on PPD antigen, but later the antigen was replaced by MTB-specific antigens, such as early-secreted antigenic target (ESAT-6) and culture filtrate protein (CFP-10) [71]. IFN-*γ* assays do provide an improved diagnosis of TBL; however, since they detect the presence of the host's CMI response towards MTB antigens, fresh blood samples are required for the test. Inability to differentiate between TBA and TBL, poor reproducibility, and reduced efficacy in children are additional problems of the CMI-based diagnostic tests [72]. In developing countries, TST is still preferred over IFN-*γ* assay due to its lower cost but suffers from low efficacy in children, poor reproducibility, and reduced diagnostic accuracy for TBL [72–74].

### 2.4. Humoral-Immune-Response-Based Testing

 In circumstances where medical resources (facilities and health care providers) are limited, serodiagnostic methods for detection of anti-MTB antibodies have some advantages (i.e., simplicity, low cost, and requirement of minimum medical resources) over aforementioned diagnostic methods [75]. Several target molecules (antigens) have been used to detect the humoral responses (anti-MTB antibodies) in TB patients. Early assays used PPD or other crude extracts as antigens for capturing anti-MTB antibodies; however, these showed poor specificity as dominant antibody responses are against cross-reactive antigens (i.e., antigens commonly found in MTB and also in other mycobacteria) [24]. As molecular techniques have improved, many antigens have been evaluated in serological tests, especially in the format of the enzyme-linked immunosorbant assay (ELISA). Some major antigens used in such tests are discussed below.

 Antigen 5, also known as 38 kDa antigen, is the best studied and most available antigen for MTB diagnosis due to its expression in the *E. coli* system. Many attempts to develop an improved serological assay for TB have used this antigen [30, 76]. Early studies reported 89% sensitivity and 100% specificity in TBA patients [31]. Later studies showed even higher sensitivity and demonstrated a correlation between antibody level and bacterial load [77–80]. As summarized in a review article [81], detection of antibodies against Antigen85 complex in ELISA formats achieves 50% sensitivity; however, this complex is highly cross-reactive and often generates false-positive results in individuals infected with atypical mycobacteria. A cell wall component, called a cord factor (trehalose-6,6′-dimycolate), used as antigen in ELISA format achieved 84% sensitivity with 100% specificity [32]. However, in a subsequent study, it was shown that anticord factor antibodies decline after antituberculous chemotherapy, which makes it difficult to determine the status of the infection in such patients [33]. Studies of the serodiagnostic potential of ESAT-6 [34, 35] and CFP-10 [34, 35, 39, 40] have also been conducted. One showed low sensitivity (67%) and specificity (51%) for ESAT-6 [34]. Low sensitivity (48–63%) also has been reported for CFP-10 [34, 82]. In high incidence areas, neither ESAT-6 nor CFP-10 antigens are useful in differentiating between TBA and TBL [34]. Another antigen, Kp 90, has been used in ELISA format to detect IgA antibodies against the protein; the results, when compared with NAA and other serological assays, indicated that anti-Kp 90 antibodies were detected in 78% of serum samples and 69% of samples from synovial, cerebrospinal, and abscess body fluids [41].

 Antigen 60 (A60) is the main thermostable component of PPD [83, 84]. Many studies have used this antigen and found almost 100% specificity [42], with sensitivity ranging from 68 to 91% [43, 85]. Unfortunately, this molecule has also been found in nonpathogenic *Nocardia* and *Corynebacterium* species [83]. A 30 kDa antigen (isolated from a culture filtrate of MTB, Antigen 85B) was used in dot immunoassay, and the result was compared with that of standard plate ELISA. The specificities of the dot immunoassay and ELISA were 92% and 97%, respectively, and the sensitivities in the assays were 69% and 78%, respectively [44]. Further studies showed that this antigen not only diagnosed TBA but also detected the nonprotective immune response of a healthy household contact group [86].

 Malate synthase (MS), a 81 kDa protein (present in MTB culture filtrates, cell wall, and cytoplasmic subcellular fractions) is an enzyme of the glyoxylate pathway used by MTB during intracellular replication in macrophages [50]. Studies with an MS-based assay have shown a sensitivity of 73% and specificity of 98% in smear positive patients, suggesting that MS is a potential candidate for TB diagnosis [82, 87]. The cell wall of MTB also contains lipoarabinomannan (LAM); however, its use as antigen in diagnostic tests is limited due to immune complex formation [3]. LAM antigen is found in urine of TBA patients, and tests based on detecting the LAM in urine samples have been developed [46, 88, 89].

 Steingart et al. conducted an intensive meta-analysis of 67 studies published in 1990–2006 on commercial serological tests for TBA (e.g., Detect-TB, and a-TB ELISA, ICT TB test) [75]. Antigens used in the commercial tests include Antigen 60, 38-Kda protein, LAM, and Kp-90. The meta-analysis revealed that estimated diagnostic sensitivities (0–100%) and specificities (31–100%) in the studies were inconsistent and imprecise, which is consistent with a WHO report in 2008 [90].

 In patients coinfected with HIV and MTB, the level of antibody production to TB antigens differs from that of HIV-negative TB patients. For example, an ELISA based on MS/MPT51 antigens showed positive reactions in approximately 80% of HIV-positive, TB-positive patients and in 42% of HIV-negative, TB-positive patients [51]. Wanchu suggested that better diagnosis of TB will require a focus on development of multi-antigen-based tests and identification of novel MTB proteins that increase in HIV patients [91].

### 2.5. Point-of-Care Diagnosis and Future Directions

 The studies described above indicate the need for an improved diagnostic test that is better able to differentiate the three forms of TB infection and to diagnose TB in the presence of HIV infection. Furthermore, since most deaths due to TB occur in developing countries that lack proper laboratory facilities and specialist training, it is important to develop a simple, rapid, and cost-effective test. The Xpert MTB/RIF assay has been recently used as point-of-care diagnosis for MDRTB and drug-sensitive TB [92, 93]. Although simple to perform and highly sensitive, this assay is costly [94]. McNerney and Daley have summarized the importance of point-of-care diagnosis [95] and suggest three important areas in which progress should be made to achieve better point-of-care for TB. The first is through identification of biological, metabolic, and pathogen-derived markers that will assist in understanding the disease. The second is the development of effective technologies like immunochromatography and nanotechnology. The third is to better understand the economical and logistic constraints on the implementation of new tests [95]. In summary, there is an urgent need to develop a lab-free diagnostic device for TB that will decrease disease transmission rate, reduce death rates, and permit faster initiation of treatment.

## 3. Bovine Tuberculosis

### 3.1. Background

Bovine tuberculosis (bTB), caused by *Mycobacterium bovis *(MB), is an infectious, chronic but progressive disease characterized by the formation of granulomatous lesions with varying degrees of necrosis, calcification, and encapsulation [11]. MB is known to infect and cause tuberculosis in a wide range of wild animals, livestock animals, and humans. Although bTB has been mostly eradicated in the livestock industry of developed countries, the disease in wildlife still poses a risk to livestock, tourism economy, and wildlife conservation [11]. Infected wildlife species include white-tailed deer (*Odocoileus virginianus*) in several states of the USA, Eurasian badgers (*Meles meles*) in Great Britain and the Republic of Ireland, and brushtail possums (*Trichosurus vulpecula*) in New Zealand [6]. Global economic losses from bTB total US$ 3 billion annually [5]. In the USA, US$ 40 million and in Great Britain *£*100 million were spent on bTB management in the year 2008-2009 alone [5]. In developing countries, bTB still causes serious concerns not only for wildlife, but also for public health, food safety, and the economy of livestock industries. More accurate diagnosis of bTB would reduce the unnecessary sacrifice of healthy animals and would also help to more effectively control bTB. At present, postmortem diagnosis based on examination of gross lesions, followed by histopathology and culture, is widely used for surveillance of bTB in wild animals, but this method is time-consuming and cannot diagnose an early infection [96].

### 3.2. Cell-Mediated Immune-Response-Based-Testing

The ante mortem diagnostic method currently prescribed by OIE is the intradermal tuberculin skin test (TST) [97]. The TST is by far the most effective test used in the eradication of bTB in the developing countries. The test is performed by injecting a small volume of bovine tuberculin in the skin of the animal and palpating a change in the thickness of the skin at the site of injection after 48–72 hours. The tuberculin used in most of the countries is derived from cultures of MB AN5, a field strain isolated in England circa 1948 [5, 26, 96]. The TST is, however, susceptible to causing false-positive reactions due to exposure of some animals to environmental mycobacteria such as *M. avium* and MAP [96, 98–100]. TST can also cause false-negative reactions due to immunosuppression, desensitization towards tuberculin, subpotent use of tuberculin, and lengthy exposure to a field strain [96]. Steps have been taken to improve specificity by using specific antigens, such as ESAT-6 [101] and a cocktail of ESAT-6/CFP-10/MPB83; however, these studies still need to be validated at a larger scale [5].

 Revisiting the animal after 2-3 days application of the TST to check their reaction is labor intensive (and usually impractical for free-ranging wildlife). The alternative IFN-*γ* assay is an *in vitro* blood test based on measuring the CMI response of the animals infected with MB [102]. The IFN-*γ* assay is usually performed using PPD as antigen, although recent studies have evaluated ESAT-6 and CFP-10 [103–106]. A problem with the IFN-*γ* assay is that it is a costly process that requires well-trained personnel to carry out the test [26, 107]. Bacteriological culture of clinical samples (i.e., milk, blood, nasal swab, and cattle tissues) is considered to be the gold standard for bTB diagnosis but, the test requires a minimum of several weeks [96, 108]. Nucleic acid amplification methods (e.g., PCR) have been also used for bTB diagnosis, but these methods are costly, less sensitive than the bacteriological culture test and again require a trained technician to perform the test [96, 108–110].

### 3.3. Humoral Immune-Response-Based Testing

 Another type of immunological test is based on detection of humoral immune response (i.e., antibody production). The major advantages of the antibody-detection tests are that they are inexpensive and relatively easy to perform. However, low sensitivity of the antibody-detection tests remains a concern. Several attempts have been made to develop ELISA tests for detection of antibody response against MB infections. PPD was used as an antigen to measure antibody response in animals with MB infection [111, 112], but the cross reactivity of PPD with closely related mycobacterial species has always been a concern. Auer [113] used a sonicated preparation of MB as antigen and reported low specificity [113]. Further studies used a specific protein isolated from MB bacillus Calmette-Guerin (BCG) strain, MPB70, as an antigen for developing assays for the diagnosis of bTB. The use of MPB70 achieved better specificity (96.4%) but had poor sensitivity (18.1%) [114–116]. Ag85 complex consists of the major secretion products of MB BCG strain and has 3 major components: 85A (31 kDa), 85B (30 kDa), and 85C (31.5 kDa). This complex is strongly immunogenic and has been used for the development of assays to diagnose TB and bTB. However, low sensitivity was reported from studies using Ag85 in ELISA format and attributed to false-positive reactions caused by infections with environmental mycobacteria [54, 81, 115]. MPB83 has been used as antigen in many studies and is a very promising candidate for bTB serodiagnosis [53]. As discussed in the TB section, LAM, ESAT-6, and CFP-10 have also been used as antigens to detect antibody response against MB [117–122]. Further, as molecular biology tools have improved, recombinant proteins have come to be used as antigens for diagnosis of bTB. Since recombinant proteins can be produced at large scale, they are cost-effective and provide consistency in their quality as diagnostic antigen [55, 123, 124].

### 3.4. Point-of-Care Diagnosis and Future Directions

One of the promising antibody-based detection assays, Multi-Antigen Print Immuno-Assay (MAPIA), is based on immobilization of antigens onto nitrocellulose membranes by semiautomated microspraying, followed by standard chromogenic immune development. This serodiagnostic test uses a cocktail of multi-antigens, such as MPB83/70, ESAT-6, and CFP10 [36]. In a recent study, seroreactivity with MPB83 in deer was 89%; however, MAPIA showed that 26% of these were false positives [37]. Based on these MAPIA results, a new version of an immunochromatographic test format for rapid diagnosis of MB infection, called rapid test (RT), was developed using colloidal gold conjugated to protein A. RT uses recombinant proteins of MPB83 and TBF10 printed onto a membrane either separately as two bands or as a combination of the two antigens in one test line [56]. Diagnostic sensitivity of the RT in experimentally infected deer was 79%, whereas that in naturally infected deer was 67% [37]. Jaroso et al., [125] compared the RT with the comparative cervical skin test (CCT) and found similar sensitivities of 80.1%. They also found that by combining the results from both RT and CCT, the sensitivity was 100%. It was suggested that the combined uses of RT and CCT would maximize sensitivity of bTB detection [125]. Some recent studies have concluded that ESAT-6 and CFP10 (used either individually or as cocktail) are better candidates for diagnosis of bTB [126–128].

 MAPIA and RT can be conducted in field situations and so can contribute to effective testing/control of bTB, especially in wild animals. However, interpretation of the test results in MAPIA and RT relies on observation of color development on a strip, which may vary depending on examiners. Higher accuracy and consistency could be achieved via a lab-free diagnostic device that outputs numerical data based on level of antibody binding to MB antigen(s). Further, as we discussed above, effort needs to be directed towards identifying a better antigen (or a combination of antigens) to further improve diagnostic sensitivity and specificity.

## 4. Johne's Disease

### 4.1. Background

Johne's disease (JD) or paratuberculosis is a chronic infectious enteritis of domestic and wild ruminants, causing reduction in milk production, malnutrition, weight loss, and eventually death [129, 130]. JD is prevalent worldwide and has a significant impact on global animal husbandry. In the USA, the causative agent of JD, *Mycobacterium avium* subsp. *paratuberculosis* (MAP), is found in 68% of dairies [8], with average herd-level prevalence of JD estimated to be 22%. The annual loss in the US dairy industry caused by MAP infection has been estimated at $220 million [9]. Economic losses associated with JD arise from decreased milk production, reduced fertility, and higher rate of culling [131]. In addition to the economic impact of JD to dairy industry, it is possible that MAP plays a role in Crohn's disease, which is an inflammatory bowel disease in humans [132]. These economic and possible health concerns create an urgent need for improved control of JD. As no practical treatment is available for JD, a better understanding of the transmission, detection, and management of the disease are the recommended procedures for its control [133].

### 4.2. Bacterial Culture and Cell-Mediated Immune-Response-Based Testing

 Diagnostic tests to detect infection with MAP can be categorized as those that identify the organism and those that identify the immunological response to the organism. Fecal culturing for MAP using Herrold's egg yolk medium (HEYM) has been considered as a gold-standard test for JD diagnosis; however, it takes as long as 16 weeks to see an observable growth. Other approaches, such as the use of BACTEC radiometric liquid culture [134, 135] and MGIT culture medium [136], have been examined to reduce the culture time but these approaches require a specialist and are relatively expensive. Polymerase-chain-reaction- (PCR-) based diagnosis using IS900 insertion sequence [137], HspX [138], or F57 DNA fragment [139], on the feces of suspect animals can also be used. This PCR-based approach is much faster but is less sensitive than the culture test because PCR reaction can be inhibited by substances in the feces. Animals develop both CMI and humoral responses against MAP. A CMI-based diagnostic test, the IFN-*γ* assay, has been evaluated using blood samples of experimentally infected cattle. The study demonstrated that the IFN-*γ* assay could detect MAP infections in early stage of JD [140, 141]; however, IFN-*γ* assay is affected by antigen stimulation and blood sampling-storage conditions [142, 143]. This suggests that the IFN-*γ* test requires further optimization.

### 4.3. Humoral Immune-Response-Based Testing

 Three different tests are used to measure antibody response in JD: complement fixation, agar gel immunodiffusion, and ELISA. The complement fixation and agar gel immunodiffusion tests both suffer poor sensitivity [144], and so a recent report has suggested that ELISAs are the best of the three methods for controlling JD in dairy and beef herds [133]. Diagnoses of JD using ELISA have been reported in many previous studies using different antigens [28, 29, 48, 63, 141, 145–151]. The antigens used in these studies have used protoplasmic antigen (PPA) [28, 29, 146, 147, 149, 150], lipoarabinomannan (LAM) [48], culture filtrate of MAP [63], and MAP proteins-1152 and 1156 [151] for testing antibodies against MAP. Beam et al. described a crude antigen mixture termed PPA, which is prepared by thorough physical disruption of mycobacterial bacilli followed by removal of cell debris and cell wall components [152]. Although many investigators have prepared PPA using various preparation protocols, it contains proteins very similar to proteins commonly found in closely related mycobacteria species. LAM is one of the components of the cell wall of mycobacteria species [120], and its core structure is shared among mycobacterial species [153].

 Sweeney et al. tested milk and serum samples in an LAM-based ELISA to detect antibodies for JD diagnosis and found that sensitivity and specificity of the ELISA were similar regardless of the tested samples (i.e., milk and serum) [48]. McKenna et al. [49] compared diagnostic performance of PPA-based ELISA and LAM-based ELISA using fecal culture test as a gold standard. Sensitivity and specificity of the PPA-based ELISA were higher than that for the LAM-ELISA [49]. PPA and LAM both contain structures common in mycobacterial species, so the use of these molecules as diagnostic antigen can cause false-positive reactions in animals infected with environmental mycobacteria other than MAP [154].

 Bannantine et al. tested 18 purified recombinant proteins in ELISA format for serodiagnosis of ovine paratuberculosis. They found that MAP proteins 0862 and 3786 demonstrated the strongest antibody response and MAP protein 2116c the weakest [58]. Shin et al. used culture filtrate of an MAP strain, JTC, in ELISA format for JD diagnosis and named the method JTC-ELISA [63]. JTC-ELISA showed significantly higher sensitivity (56.3%) than that of commercial ELISA tests (28–44%) and performed effectively on both serum and milk samples. As mentioned above, the recommended control measure for JD is testing herds by ELISA methods but the current ELISA tests have low sensitivity (28–44.5%) [29]. We have previously reported that the surface antigens of MAP are capable of detecting anti-MAP antibodies in serum at early stages of JD [59, 60]. Since mycobacteria are known to express species-specific lipidic molecules on their surface, surface antigens were extracted by gently mixing MAP with various organic solutions and tested for antibody binding in ELISA format [61]. Antigens extracted from MAP by using 80% ethanol showed the greatest differentiation between antibody binding in JD-negative and JD-positive serum samples [61]. An ELISA test developed using the ethanol extract has been named ethanol vortex ELISA (EVELISA). The results from EVELISA showed that 98.4% of the JD-positive samples had higher antibody binding levels than those of JD-negative samples, whereas the percentage of positive antibody binding in a commercial ELISA test was 50% [61]. By using thin layer chromatography, species-specific lipidic molecules were detected in the ethanol extract (unpublished data). Eckstein et al. reported that species-specific antigenic lipopeptides (e.g., Para-LP-01) exist on the surface of MAP [155], and the high sensitivity of the EVELISA may be attributed to these lipopeptides.

 ELISA, as well as other methods for JD diagnosis, needs to be conducted in diagnostic laboratories employing staff with expertise in microbiology, molecular biology, and immunology. This requires a labor-intensive process involving collecting samples into proper containers, indexing, packing, and shipping. Furthermore, cost per sample is relatively high—testing a sample by current fecal culture, PCR, and ELISA tests cost $16–19, $25, and $5-6, respectively, and this does not include costs associated with site visits and sample collections and shipping [133]. Because of the labor and cost for the current JD diagnosis, screening of cattle herds for JD is generally conducted at an interval of 6–12 months. During this interval, nonshedding animals can become shedders and low-shedding animals can become high shedders, thereby spreading MAP infection widely in the herd. This relatively long time interval between JD screening tests, in combination with low sensitivity of current diagnostic tests, may have been a reason that MAP infections remain so widespread in the US dairy and beef industries.

### 4.4. Point-of-Care Diagnosis and Future Directions

 Controlling JD requires a better understanding of the spread of MAP in a dairy herd, which can be achieved by continuous monitoring of the infection using a lab-free diagnostic device. For development of a lab-free diagnostic device, microfluidic technology has begun to be employed in the last decade [15]. Microfluidic devices are state-of-the-art tools for biochemical and immunological analysis that have high sensitivity, require only short periods of time, small amounts of reagents, and do not require an expert operator [13, 14, 156]. In our recent study, we developed a prototype of lab-free diagnostic device for JD by using a microfluidic technology and the antigen used in the EVELISA test [157]. The device is composed of microfluidic channels/chamber with electrodes, light source for fluorescence excitation, and light detector. The EVELISA antigen was immobilized in the microchannel and reacted sequentially with bovine serum sample and fluorescently labeled secondary antibody. Liquid flow was controlled by applying AC signals to the electrodes in the microchannel. Further, antibody-antigen interaction was accelerated by creating liquid vortices by applying AC signals to the reaction chamber. The major advantages of this system are its low cost, ultraportable, and disposable immunoreactions chip, and the ability to detect antibodies within 20 min [157].

## 5. Conclusion

Among the diagnostic methods used for TB, bTB, and JD, serological methods have some compelling advantages that include short turn-around time, simple procedure, and low cost. However, as summarized in Table 1, previous reports on serodiagnosis indicated a lack of diagnostic accuracy and/or insufficient-tested samples for validation of the estimated diagnostic accuracy. The low diagnostic accuracy of the current serodiagnosis for the mycobacterial infections may be due to the false-positive reactions (causing low specificity), arising from exposure of some tested individuals to other nonpathogenic environmental bacteria. Recent studies have indicated that the use of multiple species-specific antigens may improve diagnostic accuracy of the serodiagnosis of the mycobacterial diseases. Some nonprotein molecules (cord factor and lipoarabinomannan) were also evaluated for serodiagnosis of mycobacterial infections. Since mycobacteria are known to produce a variety of species-specific non-protein molecules, further efforts to identify non-protein diagnostic antigens may be a useful contribution to the development of more specific tests for TB, bTB, and JD.

Most, if not all, of the current diagnostic tests for mycobacterial infections are carried out in a diagnostic laboratory, causing cost for sample processing and/or long turn-around time. Lab-free diagnostic devices would be valuable in understanding the epidemiology of the mycobacterial infections and would facilitate their control. The emergence of new technology, microfluidic lab-on-a-chip (LOC), holds considerable promise for accelerating the development of lab-free diagnostic devices for these mycobacterial infections. LOC refers to miniaturized devices that can perform single or multiple laboratory procedures on a chip with a footprint of only a few inches in size [158]. Various LOCs have been developed for biochemical assays, detection of small particles (cells and bacteria), single-cell analysis, immunoassays, and so forth. Because of its small size and capability of automation, the technology offers opportunities for the development of point-of-care diagnostic devices for various diseases and physiological conditions. In the last decade, LOC technology has been employed for development of antibody detection assays [159, 160]. The principle of the immunoassay is same as conventional serological tests—detection of antibody binding to immobilized diagnostic antigen. However, whole assay processes (antibody reaction, washing, and detection) are carried out in a microfluidic system (microchannels). Liquid flow in the microchannel is controlled by electric fluid handling, pressure-driven fluid handling, or passive capillary force fluid handling [160]. Detection of antibody binding in LOC is based on either optical or nonoptical detection methods [159]. The most common types of optical detection systems are fluorescence detection and surface plasmon resonance. Fluorescence detection is highly useful technique due to its high sensitivity and the ease of integrating a label to the marker [159]. Surface plasmon resonance technology is based on measurement of the change in plasmon mode due to binding of biomolecules (antibody) to the surface (immobilized antigen) [161]. The nonoptical detection system is based mainly on measurement of change in the electrochemical properties due to molecular interactions on the reaction surface. This approach (i.e., a label-free sensor) does not require cumbersome detection system and therefore makes the LOC device relatively small and inexpensive [161]. Although development of lab-free diagnostics for mycobacterial diseases is in its infant stage, a recent study demonstrated the detection of MTB using fluorescent markers [95, 162]. Also, we recently reported that a prototype of LOC-based system could detect antibodies in JD-positive serum in 20 min [157]. Further, the system was converted to a label-free system using an electrochemical detection, reducing the detection time to 2 min (unpublished data).

 A combination of species-specific (multi) antigens and LOC technology may lead to development of an accurate on-site (in-field) diagnostic device and thereby contribute to effective control of mycobacterial infections.

## Figures and Tables

**Table 1:** Summary of humoral immune response based assays.

Target antigen	MTB/MB/MAP	Method of testing	Se (%)	Sp (%)	*P*	*N*	Species tested	Reference
PPD	MTB	ELISA	89	87	18	83	Human	[24, 25]
PPD	MB	ELISA	68–95	96–99	120	223	Cattle	[26, 27]
PPD	MAP	ELISA	29–72	99	359	2094	Cattle	[28, 29]
Antigen 5 (38 kDa)	MTB	ELISA	89	94–100	82	30	Human	[30, 31]
Cord factor	MTB	ELISA	81–84	96–100	65	66	Human	[32, 33]
ESAT-6	MTB	ELISA	67	51	100	100	Human	[34, 35]
ESAT-6	MB	ELISA	49–59	84–95	522	1489	Cattle	[36–38]
ESAT-6	MB	MAPIA	67	98	9	98	Deer	
CFP-10	MTB	ELISA	48–63	51–71	100	100	Human	[34, 39, 40]
CFP-10	MB	ELISA	49–59	84–95	522	1489	Cattle	[36–38]
CFP-10	MB	MAPIA	56	99	9	98	Deer	
Kp90	MTB	ELISA	78	82	51	71	Human	[41]
Antigen 60	MTB	ELISA	68–91	100	337	131	Human	[42, 43]
30 kDa antigen	MTB	ELISA	84	96.7	175	150	Human	[44, 45]
LAM	MTB	ELISA	17.8	87.7	47	153	Human	[27, 46–49]
LAM	MB	ELISA	60	na	120	—	Cattle	
LAM	MAP	ELISA	66	88	167	216	Cattle	
MS	MTB	ELISA	73–75	97-98	35	17	Human	[50–52]
MPT51	MTB	ELISA	80	na	53	—	Human	[51]
MPB70	MB	ELISA	73	88	120	223	Cattle	[27, 36–38, 53]
MPB70	MB	MAPIA	44	100	9	98	Deer	
Antigen 85 complex	MB	ELISA	48	89	208	54	Cattle	[54, 55]
MPB 83	MB	ELISA	49–59	84–95	522	1489	Cattle	[36, 53, 56]
MPB83	MB	MAPIA	89	99	9	98	Deer	[36–38, 53, 56, 57]
MPB83	MB	RT	60	96	25	25	Cattle	
MAP proteins 0862 and 3786	MAP	ELISA	81	na	11	—	Sheep	[58]
Ethanol extract	MAP	ELISA	97.4	100	64	38	Cattle	[59–62]
JTC	MAP	ELISA	56.3	99	444	412	Cattle	[63]

Se, Sensitivity; Sp, Specificity; *P*, no. of positive samples tested; *N*, no. of negative samples tested.
